# Death of Dr. William P. Dewees

**Published:** 1841-06

**Authors:** 


DEATH OF DR. DEWEES.

The Philadelphia papers announce the death of Dr. William P. Dewees, formerly Professor of Obstetrics in the University of Pennsylvania. It took place on the 18th instant, in the seventy-fifth year of his age. A few years ago Dr. Dewees resigned his chair, on account of declining health, and went to Alabama; he resided for a while at Mobile, then in New Orleans, and finally returned, about twelve months ago, to Philadelphia, where he has closed a life of great usefulness among his former friends. The nature of his last illness is not stated. As a practitioner of midwifery, he enjoyed for many years the highest confidence of the community, and his business in that branch of medicine was undoubtedly much more extensive than ever fell to the lot of any one individual in America. Indeed, such was his reputation at one time that very few of the more respectable dames of the city of "brotherly love" were willing to be delivered without his aid. In all difficult cases his counsel was invariably sought by his confreres.

Dr. Dewees was a voluminous writer. His works on midwifery, and the diseases of women and children are every where known, but the question as to their real merit is not definitively settled. Their chief faults are that they are exceedingly verbose, highly inelegant in their style, wholly deficient in pathological details, and full of the grossest dogmatism. In this latter respect they form a striking contrast with the writings of the late Professor Eberle, which are as modest and unassuming, as those of Dr. Dewees are bold, and authorative. The tendency of both is decidedly injurious to the true interests of medical science. As a teacher Dr. Dewees occupied, we believe, a high rank. For many years he was adjunct to Professor James, on whose death he was elevated to the vacant chair.

